# Design of AI-driven microwave imaging for lung tumor monitoring

**DOI:** 10.1038/s41598-025-20566-w

**Published:** 2025-10-01

**Authors:** Adarsh Singh, Sandip Paul, Sreetama Gayen, Bappaditya Mandal, Debasis Mitra, Robin Augustine

**Affiliations:** 1 Department of Electronics & Telecommunication Engineering, Indian Institute of Engineering Science and Technology, Shibpur, India; 2 Division of Solid State Electronics, Department of Electrical Engineering, Ångström Laboratory, Uppsala University, 75121 Uppsala, Sweden; 3 Department of Electronics and Telecommunication Engineering, Kolaghat Government Polytechnic, West Bengal, India

**Keywords:** Microwave Imaging, Lung Tumor, AI, XGBoost, CNN, Health care, Engineering

## Abstract

The global incidence of lung diseases, particularly lung cancer, is increasing at an alarming rate, underscoring the urgent need for early detection, robust monitoring, and timely intervention. This study presents design aspects of an artificial intelligence (AI)-integrated microwave-based diagnostic tool for the early detection of lung tumors. The proposed method assimilates the prowess of machine learning (ML) tools with microwave imaging (MWI). A microwave unit containing eight antennas in the form of a wearable belt is employed for data collection from the CST body models. The data, collected in the form of scattering parameters, are reconstructed as 2D images. Two different ML approaches have been investigated for tumor detection and prediction of the size of the detected tumor. The first approach employs XGBoost models on raw S-parameters and the second approach uses convolutional neural networks (CNN) on the reconstructed 2-D microwave images. It is found that the XGBoost-based classifier with S-parameters outperforms the CNN-based classifier on reconstructed microwave images for tumor detection. Whereas a CNN-based model on reconstructed microwave images performs much better than an XGBoost-based regression model designed on the raw S-parameters for tumor size prediction. The performances of both of these models are evaluated on other body models to examine their generalization capacity over unknown data. This work explores the feasibility of a low-cost portable AI-integrated microwave diagnostic device for lung tumor detection, which eliminates the risk of exposure to harmful ionizing radiations of X-ray and CT scans.

## Introduction

In recent times, advancements in biomedical research continue to shed light on complex health conditions that demand urgent attention. Respiratory disorders or lung diseases, in particular, have increased globally, highlighting the need for improved diagnostic and treatment strategies. The lung diseases include a variety of conditions that affect how the lungs work. These diseases can appear in different ways, making it harder for people to breathe and impacting their overall health. While the symptoms may seem similar to the flu, lung diseases are often much more serious and can lead to dangerous outcomes if not treated quickly. This makes early diagnosis very important to prevent serious complications^1^.

According to WHO^2^, lung cancer stands as the primary contributor to cancer-related fatalities globally, presenting the highest mortality rates across both male and female populations. Lung cancer is a malignant neoplasm that originates in the tissues of the lungs. It is characterized by the uncontrolled growth of abnormal cells in the lung tissues, which can form tumors and interfere with the normal functioning of the lungs.

According to the mortality data provided by IARC^3^, lung cancer emerges as the foremost contributor to cancer-related deaths, accounting for around 1.8 million fatalities out of the total 9.6 million cancer deaths in 2022. The same study reveals that among males, lung cancer leads with 1.2 million recorded deaths, securing the highest position. In the female population, lung cancer follows breast cancer as the second-highest cause of cancer-related deaths, with 0.58 million fatalities. The development of lung cancer is often associated with risk factors such as smoking (85% of all cases), exposure to secondhand smoke, radon exposure, and a family history of lung cancer^2^. Lung cancer is histologically categorized into two primary classifications: small-cell lung cancer (SCLC) and non-small cell lung cancer (NSCLC). SCLC constitutes around 15% of lung cancer cases, whereas NSCLC accounts for approximately 85%^4^. NSCLC can be categorized into four major stages based on tumor size and the spread of the cancer. The chances of recurrence of lung cancer are significantly high; generally, a more advanced stage indicates a wider spread of cancer and an increased likelihood of its recurrence (5–19% for stage I, 11–27% for stage II, 24–40% for stage III, and more than 63% for higher stages)^5^. With lung cancer being the foremost threat among cancers, its elevated risk of recurrence underscores the importance of early detection and consistent post-treatment surveillance. Also, lung cancer is associated with an abnormal growth of tumors in the lung; so detection of lung cancer primarily involves detection of tumor growth in the lung and subsequent medical tests for malignancy.

The current screening methods employed in medical practice, namely X-rays and CT scans, which are extensively utilized for the purpose of identifying lung tumors and other lung disorders, are associated with a number of drawbacks and limitations^6^. The utilization of certain equipment in monitoring processes frequently involves the utilization of bulky and costly apparatus. Moreover, the utilization of ionizing radiation, which is an integral component of such equipment, can potentially pose risks to the human body if exposed for prolonged durations repeatedly.

Consequently, this renders the equipments unsuitable for continuous monitoring purposes. In the realm of disease detection, the utilization of microwave imaging techniques has emerged as a promising avenue for overcoming the limitations associated with conventional methods^7^. By utilizing the capabilities of microwave technology, researchers and medical professionals can potentially surmount the challenges that have impeded accurate and efficient disease detection in the past^8^. Microwave imaging (MI) has the potential to revolutionize healthcare by providing a non-invasive, cost-effective, and reliable method for diagnosing a variety of diseases. This imaging technique uses low-power electromagnetic waves to capture insights of anomaly in internal tissues, making it particularly appealing for applications where traditional imaging methods, such as X-rays or CT scans, may pose radiation risks or require expensive equipment. MI operates primarily in the microwave frequency range, which allows for better tissue penetration and enhanced contrast between different biological structures due to the varying dielectric properties of tissues, such as fat, muscle, and tumors^9^.

A key focus of research in microwave imaging is its potential for tumor detection, particularly in the brain, breast, and lungs. While effective, traditional imaging techniques like mammography or MRI have limitations such as exposure to ionizing radiation, high costs, and limited accessibility in resource-constrained settings. Microwave imaging offers a promising alternative by being non-ionizing and less expensive while also providing high sensitivity to differences in tissue composition. This is particularly important for early-stage tumor detection, where minor changes in tissue properties may be difficult to detect with conventional methods. Despite these advantages, microwave imaging still faces several challenges, such as the relatively low resolution compared to conventional imaging techniques. Also, microwave wavelengths are larger than the fine anatomical structures being imaged, which can result in lower spatial resolution. Further, to address this issue, advanced signal processing algorithms and image reconstruction techniques are being developed to enhance the clarity and accuracy of MI results. Furthermore, different techniques like time-domain, frequency-domain, and machine learning-based algorithms have been introduced to improve image quality and diagnostic accuracy, particularly in distinguishing healthy tissue from tumors^10^.

Another limitation of MI is its sensitivity to noise and artifacts caused by factors like body movement or interference from surrounding tissues. Since microwave signals can be scattered or absorbed by different types of tissues, it becomes challenging to isolate the specific region of interest, such as a tumor or abnormal growth. Researchers are developing robust algorithms and sensor arrays to better handle these artifacts and improve the signal-to-noise ratio, making the technology more reliable for real-time diagnostics^11^. When it comes to lung disease detection, microwave imaging holds promise but also requires further development. The lungs present a particularly challenging environment for microwave imaging due to their air-filled structure, which causes significant scattering and absorption of microwave signals. However, recent advancements in adaptive algorithms and sensor designs have shown that it is possible to achieve more accurate lung imaging by compensating for these complexities. For example, hybrid imaging techniques that combine microwave imaging with other modalities, such as ultrasound or MRI, are being explored to enhance the detection and monitoring of lung conditions, including tumors and pulmonary diseases^12^.

Previously reported microwave imaging systems for lung disease detection have primarily been externally mounted and immobile, requiring patients to visit the hospital for examination, which can be inconvenient and less preferable. One such system, designed for non-invasive detection and monitoring of pulmonary edema, consists of two linear arrays, each with eight antennas embedded inside a hospital bed where the patient must lie down^13^. Another non-portable torso scanner system features 14 complex 3D antennas in an elliptical array, placed on a movable flange. In this case, the person must stand inside the scanner system for the torso region to be scanned^14^. These traditional approaches highlight the need for more portable and convenient imaging solutions for detecting lung diseases.

The integration of microwave techniques and AI greatly improves disease detection by merging non-invasive imaging with advanced data analysis. Microwaves offer comprehensive tissue information, while AI algorithms enhance the identification of subtle irregularities, resulting in expedited and more precise diagnoses. This synergy accelerates decision-making based on AI-analyzed patterns from microwave data^15^. These approaches^16,17^ mark a major advancement in healthcare, offering greater efficiency, precision, and personalized care.

This study explores the feasibility and design aspects of an AI-based microwave diagnostic system in the form of a wearable belt that can highlight lung tumors and categorize them into NSCLC stages. The proposed approach focuses on a wearable belt having eight antennas that will generate a 2D microwave image of the torso region. Recently, with the advancement of AI, numerous architectures have been developed. However, based on the nature of the data and the size of the dataset, two different ML approaches have been chosen to investigate their performance for tumor detection and estimating its size. One of them is an XGBoost-based model that works with the S-parameter data, and the second one uses convolutional neural networks (CNN) on the 2D reconstructed microwave images. Both of these models are optimized and tested for both of the tasks, tumor prediction, and tumor size estimation. It is found that an XGBoost-based classifier with S parameters outperforms a CNN-based classifier on reconstructed images for tumor detection. Whereas, a CNN-based model performs much better than an XGBoost-based regression model for tumor size prediction.

The following section details lung cancer and its development, while the subsequent part outlines the proposed diagnostic system configuration, encompassing antenna placement, scattering parameter acquisition, and image reconstruction. Additionally, section 3 delves into the design and comparative study of machine-learning models used for tumor prediction.

The major contribution of this work contains:

- Design of a wearable antenna configuration in the form of a wearable belt over a body model available in the simulation environment for microwave imaging and sensing for lung tumor detection.
- Design of an XGBoost-based classifier for the prediction of lung tumors from raw scattering parameters obtained from antenna configuration.
- Development of a CNN-based regression model to predict tumor size.
- Performance examination of both techniques on another body model available in the simulation environment for the generalization capacity of the models over unknown data.
- Fine-tuning of naive ML models for noisy data and their performance evaluation for real-world scenarios.

This work presents a preliminary study report on the design methodology of an AI-enabled microwave imaging system for lung tumor detection and estimation of tumor size. Herein, the proposed approach focuses on design choices of the microwave sensor, on the choice of suitable machine learning models based on their performance on the simulation dataset.

## Disease and its progression

**Fig. 1:**
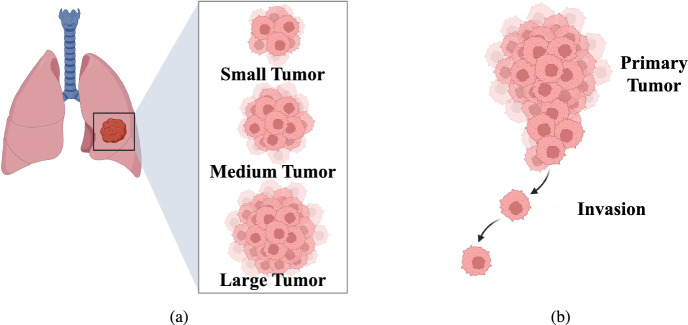
Non-small cell lung cancer (NSCLC): (**a**) tumor size and classifications, and (**b**) progression of disease. (Created in BioRender. Singh, A. (2025) https://BioRender.com/kvwc9wn).

A lung tumor is an atypical proliferation of cellular matter, occurring within the pulmonary system. There are two types of tumors: benign, which are non-cancerous, and malignant, which are cancerous. There are two main types of malignant tumors: non-small cell lung cancer (NSCLC) and small cell lung cancer (SCLC). The NSCLC is the most prevalent form, shown in Fig. 1, accounting for approximately 85% of lung cancer cases. Lung cancer can also be classified based on the affected region, including central tumors, peripheral tumors, and pleural tumors. Whereas the most prevalent subtype of non-small cell lung cancer is peripheral tumor^18^.

The progression of NSCLC can differ based on various factors, including the type of NSCLC, the stage at diagnosis, the treatment administered, and the unique characteristics of each patient. Here’s a brief overview of the progression of NSCLC:

- **Stage 0**, also known as in situ disease, indicating that the cancer remains localized and has not invaded the surrounding lung tissues or metastasized beyond the lung.
- **Early Stage (Stage I and II):** In the early stages, NSCLC is localized to the lungs and has not spread to nearby lymph nodes or distant organs. Early-stage non-small cell lung cancer (NSCLC) patients have a better prognosis than patients with advanced stages of the disease, with a larger probability of being cured. The stages and corresponding sizes of tumors are presented in Table 1. By ‘size’, the largest dimension of the tumor is denoted, and ‘radius’ denotes half of it.
- **Locally Advanced Stage (Stage III):** At this stage, NSCLC extends its reach to lymph nodes or tissues in the chest vicinity. Stage III cancers typically exhibit significant lymph node involvement while remaining localized and not spreading to distant areas of the body. Treating locally advanced NSCLC can pose greater challenges compared to early-stage disease, as surgery is not a viable option. Instead, the standard approach involves systemic therapy and radiation therapy.
- **Metastatic Stage (Stage IV):** In this stage, lung cancer can extend beyond its initial site and affect multiple areas in the other lung, the fluid surrounding the lung or the heart, or even distant parts of the body through the bloodstream. Once cancer cells enter the bloodstream, they have the potential to metastasize to various parts of the body. However, NSCLC has a higher tendency to metastasize to the brain, bones, liver, and adrenal glands. Stage IV NSCLC is further classified into two distinct substages: Stage IVA cancer has metastasized within the chest, and Stage IVB indicates the spread of cancer beyond the chest, affecting multiple locations within one organ or involving more than one organ.

**Table 1:** The stages and corresponding sizes of early-stage tumors.

**Stage**	IA	IB	IIA	IIB
Size (mm)	$\le 30$	>30 & <40	>40 & <50	$>50$
Equivalent Radius (mm)	$\le 15$	>15& <20	>20 &<25	$>25$

Generally, surgery is not a possibility for advanced stages such as III or IV lung cancers. Therefore, early detection becomes the most appropriate course of action for preventing lung cancer from going into higher stages. Even with treatment, there are instances where NSCLC may reoccur following an initial response or period of remission. Recurrence can manifest either in the lungs or in other parts of the body. Therefore, it is crucial to maintain continuous or regular assessment following treatment. Overall, the development of NSCLC can be intricate, and the strategies for treatment may differ depending on personal circumstances. Ensuring early diagnosis, accurate staging, tailored therapeutic strategies, and consistent post-treatment monitoring are crucial in enhancing the results and the well-being of individuals with NSCLC.

**Fig. 2:**
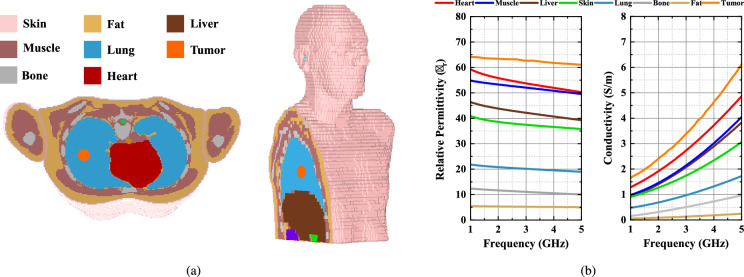
Cross-sectional views of available body model in CST Studio Suite^19^in (**a**) axial and sagittal plane (**b**) Dielectric properties of different tissues^20,21^.

Due to high water content, the dielectric properties, specifically the relative permittivity and conductivity, of tumors are much higher compared to the surrounding tissues, as demonstrated in Fig. 2. This difference in dielectric properties as a biomarker permits the development of safe and accurate microwave imaging techniques for diagnosing lung tumors.

## Methodology

**Fig. 3:**
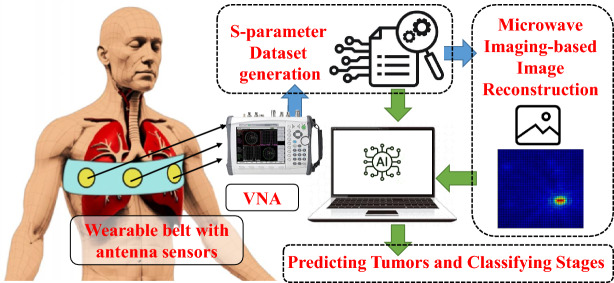
Proposed methodology for detection of lung tumors and predicting their severity stages.

The proposed system for detection of lung tumors and predicting their severity stages (as shown in Fig. 3), consists of two main subsystems: 1. a microwave sensing and imaging subsystem and 2. a machine learning subsystem for the detection of tumors and the estimation of size based on the data collected from the other subsystem.

**Fig. 4:**
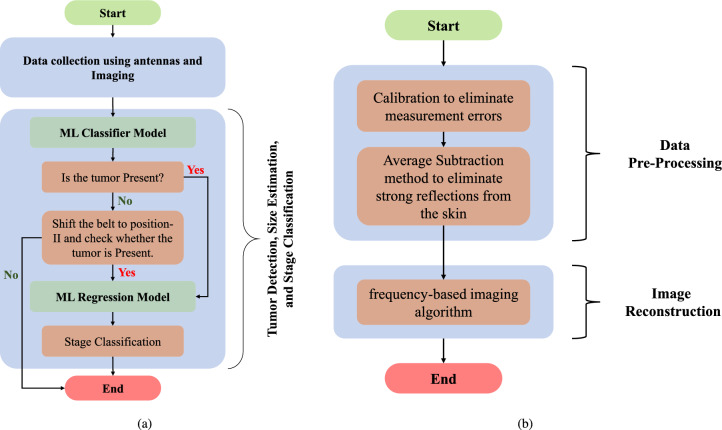
Flowchart for (**a**) the overall functioning of the proposed system, and (**b**) microwave imaging to reconstruct 2D images.

### Microwave sensing and imaging subsystem

The proposed microwave imaging system for lung tumor detection employs a sensor unit (eight antennas in the form of a wearable belt), a data collection unit (Vector network analyzer, and RF switching matrix), and a frequency-based imaging algorithm^14^. The utilized wearable antenna (Bow-tie antenna with resonance-based reflector)^22^ has already been involved in several on-body applications for sensing due to its characteristics like unidirectional radiation and wideband frequency range. The data collection unit (VNA and switching matrix) allows antennas to transmit and receive backscattered EM signals to reconstruct the image of the domain of interest (torso). Using a switching matrix^23^ with a VNA is cost-effective, enabling multiport testing with a lower-port VNA instead of an expensive high-port model. It also supports automation for faster, more efficient measurements. However, testing takes longer since ports are measured sequentially, and the switch can introduce signal losses and interference, impacting accuracy. The proposed system follows the process flow as depicted in Fig. 4

**Fig. 5:**
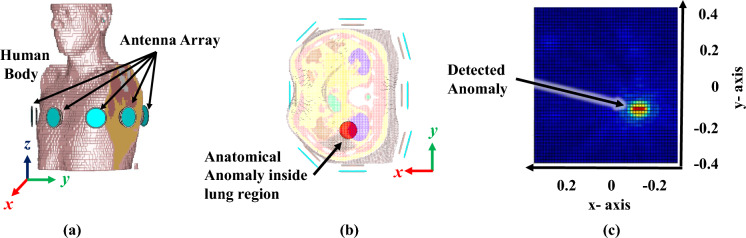
Proposed imaging subsystem: placement of antennas in the simulation environment (CST Studio Suite^19^): (**a**) Isometric view, (**b**) bottom view, and (**c**) reconstructed microwave image.

The microwave image resolution is intricately dependent on several factors, such as the number of antennas utilized, the range of broadband frequencies covered, and the density of sampled frequency points. Each of these components has a vital function in determining the accuracy and sharpness of the resulting image. When evaluating the number of antennas, it is crucial to recognize the delicate equilibrium between improving resolution and handling system complexity. In this context, for cost-effective or portable solutions, fewer antennas with machine learning-based processing can be a more accurate and practical alternative.

In the proposed work, a strategic decision has been made to employ a configuration featuring eight antennas. With this, the system strikes a balance, enabling it to capture sufficient spatial information while remaining practical in terms of limited mutual coupling between antennas, hardware integration in the form of the belt, and computational requirements. Furthermore, the choice of employing broadband operating frequency (1.5 to 3 GHz) is instrumental in ensuring versatility and adaptability in image capture. By operating across a broad frequency spectrum, the system can gather a diverse range of information, enriching the final image with comprehensive insights and details. This particular frequency range (1.5 to 3 GHz) offers an optimal balance between penetration depth and resolution^24^, which is essential for non-invasive biomedical applications. Moreover, the specific absorption rate (SAR) in this range remains low as per FCC guidelines, which ensures patient safety, especially in scenarios that require repeated or continuous monitoring^25,26^. One significant advantage of the configuration depicted in Fig. 5 lies in its adaptability for integration into wearable systems, such as a sensor-embedded belt. The compact design of the eight-antenna array, combined with its wideband characteristics, enables straightforward incorporation into wearable devices. This integration provides enhanced mobility and ease of use while maintaining high image resolution with minimal quality degradation.

**Fig. 6:**
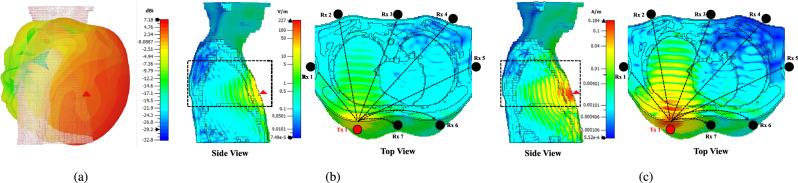
Field distributions of antenna element placed on the front side of chest: (**a**) 3-D pattern, (**b**) E-field sagittal (side) & axial (top) view, and (**c**) H-field sagittal (side) & axial (top) view (generated from CST Studio Suite^19^).

Field distributions shown in Fig. 6 demonstrate that the antenna element placed in the lower part of the chest covers the majority of the lungs, making it possible to detect the presence of any tumor in the lungs. Figure 6 (b) and (c) illustrate the distribution of electric (E) and magnetic (H) fields as they propagate through the human torso. The sagittal (side) view of the cross-sectional plane shows that the fields from the antennas effectively cover the lung region. This indicates that the placement and orientation of the antennas are suitable for capturing signals across the lungs. The axial (top) view further reveals the path of the fields from the transmitting antenna to the receiving antennas, demonstrating the travel of the EM wave through various tissues in the torso. These visualizations provide valuable insight into the propagation characteristics of the fields and the effectiveness of the antenna configuration in ensuring adequate signal coverage across the thoracic region.

**Fig. 7:**
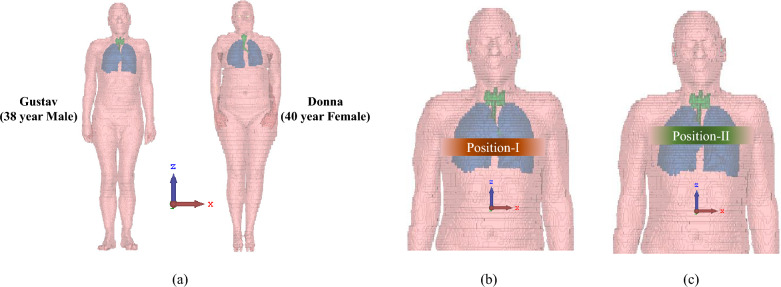
(**a**) Utilized voxel body model available in CST Studio Suite^19^; (**b**) and (**c**) Antenna belt position variations along the vertical axis.

There are two major concerns for the detection and size prediction; firstly, dealing with the error due to measurement and ambient noise; secondly, the error incurred by placement of the antenna belt far from the actual tumor location, which is generally unknown. To mitigate these issues, multiple measurements should be taken from different antenna positions. In Fig. 7, only two convenient positions are depicted, one in the lower part of the chest and another in the upper part of the chest. In an actual scenario, more than two measurements can be taken, and if the presence of a tumor is detected in any one of the measurements, it is assumed that there is a tumor. For the prediction of the size of the tumor, the maximum prediction must be taken, since it is observed in the simulation that as the antenna belt moves farther from the actual tumor location, the S-parameter values decrease significantly. However, multiple position readings are suggested as shown in Fig. 7, which would increase the likelihood of making a more robust diagnosis of tumor presence, and accurate prediction regarding the size of the tumor.

**Fig. 8:**
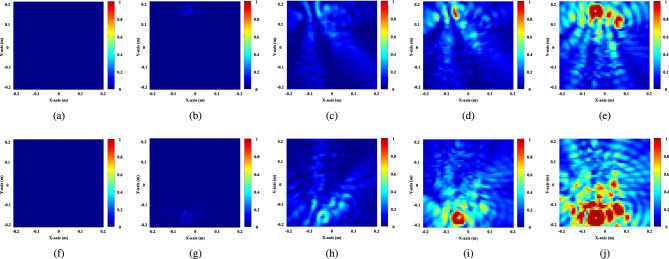
Reconstructed images from MWI for varying tumor sizes in increasing order: (**a**) to (**e**) tumor present in the left lung of sizes 0 (no tumor), 8, 20, 36, 48 mm, respectively; and (**f**) to (**j**) tumor present in the right lung of sizes 0 (no tumor), 8, 20, 36, 48 mm, respectively.

One significant step in the proposed methodology is the pre-processing of the collected data from microwave sensors (antenna system) and reconstruction of a microwave image by a frequency-based imaging algorithm (as shown in Fig. 4). In the data pre-processing step, firstly, the calibration step is performed in which S-parameters (absence of model) are subtracted by respective S-parameters (presence of model) to eliminate the clutter introduced by the imaging domain and antenna fabrication and assembly errors^27^. The chest perimeter of the patient is required as a priori information in order to calculate S-parameters in the absence of a model; Secondly, calibrated S-parameters at each frequency are averaged and then subtracted from each S-parameter. This step reduces the strong reflections from the skin tissue layer, improving the detection of abnormalities. In the further step, frequency-based imaging with a multi-static approach^14^ has been utilized to generate the 2D images. Parameters in imaging algorithm like $N_a$ (number of antennas) and $N_f$ (number of frequency points) have been updated as 8 and 500, respectively; reconstructed images are normalised to the maximum value of the healthy case as shown in Fig. 8. The proposed simulation process has been carried out on two different body models from the voxel family: Gustav, a 38-year-old male, and Donna, a 40-year-old female (shown in Fig. 7). Few samples of reconstructed images of cross-section of the Gustav body model are shown in Fig. 8, illustrating how the images change with increasing tumor sizes. It is evident from the figure that the reconstructed images effectively reveal both the presence and extent of the tumor within the complex torso medium. These models are available in the CST Studio Suite^19^. Data processing and image reconstruction were performed using MATLAB^28^ code.

### Machine learning subsystem for tumor prediction

The aim of the designed ML subsystem is twofold: first, to detect whether a tumor is present or not (framed as a classification problem), and second, to predict the size of the tumor. These two tasks are considered separately because the tumor size can be estimated accurately if the tumor lies close to the plane of the antenna belt. If the belt is placed at a location much higher or lower than the position of the tumor, the S parameters are diminished in amplitude, which hinders the accurate prediction of tumor size, but still can infer the presence or absence of a tumor.

Two approaches have been investigated for this purpose: one is based on the raw S-parameters obtained from the antenna system, and the second one takes into account the reconstructed microwave images. The designed models are trained and tested on simulation dataset. Their generalization capacity is tested on a separate dataset that contains previously unseen data obtained from a different body model. In the next section, the performance of the two types of ML models is discussed in detail.

## Investigation and design of appropriate ML models

Three sets of simulations were performed for experimentation. The first set contains a total of 140 readings (70 for each lung, i.e., 70 simulations for tumors of different sizes in each lung) obtained from Gustav’s (male) body model, corresponding to various tumor sizes varying from 2 mm to 60 mm; such that the tumor is placed at the axis of the antenna belt. The set of simulations will be referred to as Simulation-A in future references. In the second set, 20 simulations are conducted on Gustav for various tumor sizes (10 for each lung), where the position of the tumor is higher or lower than the antenna belt. This simulation will be called as Simulation-B. Simulation B is required to examine the change of S parameters of the tumor located away from the plane of the antenna belt. The final set of simulations, referred to as Simulation C, contains 10 readings from the Donna (female) body model that are used to measure the generalization capacity of the trained ML models on the unseen data.

### XGBoost model over the raw scattering parameters

All the S parameters (amplitude and phase) of the 8-antenna system are recorded for 500 frequency points over a range of 0.5 GHz to 3.5 GHz. Thus, for a particular tumor size, the raw S-parameter data dimension is 500 $\times$ 128 ($S_{11}$ amplitude, $S_{11}$ phase, $S_{12}$ amplitude, $S_{12}$ phase so on). These S-parameters contain all the necessary information regarding the presence and size of the tumor.

A very effective algorithm, named XGBoost, has been employed to process the S-parameter data and make predictions about the presence of the tumor or about its size. XGBoost (Extreme Gradient Boosting)^29^is a popular machine learning technology that is currently being hugely used for regression and classification tasks for its remarkably good and state-of-the-art performance. Also, XGBoost is being widely used in several biomedical applications^30–32^. XGBoost assimilates the decision trees with ensemble learning, where the predictions made by many decision trees are combined together to make the final prediction.

#### XGBoost for tumor detection

An XGBoost classifier is designed to predict the presence of a tumor. This is a binary classifier that differentiates the absence of a tumor or very small tumor of size $<=8mm$ (Class 0) from the presence of a tumor of size $>8mm$ (Class 1).

**Dataset construction:** The dataset for designing the classifier comprises of datapoints from Simulation A (140 simulations) and Simulation B (20 simulations). In the dataset, there are 26 data points in Class 0 and 134 data points in Class 1. In Class 0, the 26 datapoints come from tumors having sizes 2 mm to 8 mm. More datapoints can be generated using simulations, but with very small size variations, for which the S parameters would not differ significantly. It can be seen that Class 0 has a significantly lower number of data points than Class 1. This scenario is known as **class imbalance**^33^. This type of imbalance penalizes the performance of the classifier significantly. Two popular solutions to resolve this problem are: *undersampling*, where a small number of samples are collected from the majority class, and *oversampling*, where the minority class is repeatedly sampled randomly to increase the number of data points. Undersampling is simpler, but it loses important information by discarding data points. Also, lessening the dataset size hampers the training performance. One popular oversampling algorithm is Adaptive Synthetic Sampling (ADASYN)^34^. The ADASYN artificially increases the number of data points in the minority class by synthesizing more data. The algorithm identifies data points in the minority class that lie closer to the decision boundary and are difficult to classify. After applying the ADASYN algorithm, 108 new synthetic samples of the minority class are generated to make the number of Class 0 samples to be equal to the number of Class 1 samples (each to be 134).

**Construction and training of classifiers:** The balanced dataset is used for designing the XGBoost classifier. For each case, there are 64 S-parameter amplitude and phase measurements recorded over 500 frequency points ranging from 0.5 GHz to 3.5 GHz. This gives rise to 64000 ($2 \hspace{0.1 cm}\text {(amplitude and phase)} \times 64 \hspace{0.1 cm} \text {(S-parameters)} \times 500 \hspace{0.1 cm}\text {(frequency points)}$) numerical values for each case. This huge number of input values, each of which may be considered as a ”feature”, will mislead the classifier model and overfit it. To resolve this issue, each of the S parameter values (amplitude and phase) is averaged over the operating frequency range of 1.5 - 3 GHz (in this range, the variation is most prominent), thus creating a vector of length 128 S-parameter values for each tumor size.

During training, 80% of the dataset is used as a training set and 20% is used as a test set. The classifier is designed using Python’s XGBoost package^35^. During training, the model generates many trees with a predefined maximum depth and combines the predictions to construct a single prediction. In the training process, some of the parameters of the XGBoost model are adjusted heuristically to achieve the best performance on the test set. The best combination of XGBoost parameters, which is obtained heuristically by means of *grid search* over the parameter space, is as follows:

- $n\_estimators$ =10, i.e., the number of trees whose ensemble is used for classification;
- $max\_depth$ = 3, which specifies the maximum depth of each tree;
- eta = 0.3, which is the learning rate.
- subsample = 0.5, which specifies the proportion of training data considered for constructing the tree.

The performance of the trained XGBoost classifier is examined by using it to make predictions for the data points in the test set. The classifier performs really well with 100% accuracy over the ADASYN-balanced test dataset. It signifies that the distinction between class 0 and class 1 is very clear.

**Table 2:** Predicted tumor sizes by the XGBoost Regression model, CNN over intensity MW image.

**Actual Tumor Radius (mm)**	**XGBoost** Predicted Radius (mm)	**CNN on MSI** Predicted Radius (mm)
17.2	17.7	17.0
13.2	12.8	12.6
4.8	6.20	5.6
13.6	13.34	12.7
11.6	13.10	12.1
8.8	9.72	10.1
20.8	18.23	19.7
12.4	12.3	11.8
21.6	23.0	21.2
6.0	5.78	5.6

#### XGBoost for tumor size prediction

The raw S-parameters obtained from Simulation A (i.e., 140 simulations on Gustav’s body model with the tumor residing on the plane of the antenna belt) are used to predict the size of the tumor. Each data point consists of 128 S-parameter readings (amplitude and phase) taken for 500 frequency points over the range of 0.5 GHz to 3.5 GHz. Before feeding the data to the XGBoost regression model, each S-parameter (amplitude and frequency) is averaged over the operating frequency range of 1.5 - 3 GHz, where the distinction is more prominent. Thus, corresponding to each tumor size, a vector of 128 S-parameter values (amplitude and phase) is obtained. Unlike the XGBoost classifier, in the case of designing the regression model, there is no requirement for dataset balancing since the input data is almost evenly distributed.

**Construction and training of regression model:** Among the total of 140 simulations, 10 simulations are separated out as test set. From the rest, $80\%$ of the data is used for training the XGBoost tumor size-prediction model, and the rest ($20\%$) is used as a validation set for validation of the trained model. The chosen best set of hyperparameters found by means of grid search over the parameter space are as follows:

- $$n\_estimators$$ =1000,
- $$max\_depth$$ = 4,
- eta = 0.3,
- subsample = 0.5,

The rest of the parameters are kept as per their default values.

**Performance on the Test Set:** The performance of the designed classifier is examined on the *test set*, which consists of the 10 datapoints that are separated out from the original dataset before the creation of the training and validation set. This dataset is kept completely hidden from the regression model during training. The original tumor size and the tumor size predicted by the XGBoost regression model are shown in Table 2. The mean squared error for the predictions is 1.23 mm. The two-sided, two-tail t-test is performed over the actual and predicted values of test data. The observed two-tailed *p*-value equals 0.9070. With conventional criteria, it denotes that the difference between the two distributions is not statistically significant.

### CNN-based model over microwave images reconstructed from S parameters

Convolutional Neural Networks (CNN) are widely used in image-based tasks in deep learning^36–38^. CNNs capture spatial information or spatial features from an image in order to use them for different tasks. Here, we have employed two separate CNN models over the reconstructed microwave images, synthesized from S parameters, to detect the presence of a tumor as well as to predict the size. The reconstructed microwave images are color (3 channel) images having dimensions of $634 \times 496$ pixels.

#### CNN model for tumor detection

The CNN-based classifier is designed using the datasets from Simulation A and Simulation B. As described in section 4.1, the training dataset has an imbalance as there are more readings corresponding to class 1 (134 data points) than for class 0 (26 data points). This imbalance is eliminated by applying ADASYN algorithm on the reconstructed microwave images. After the application of ADASYN, synthetically generated data points are a balanced image dataset of size 268, having equal data points in class 0 and in class 1.

**Fig. 9:**
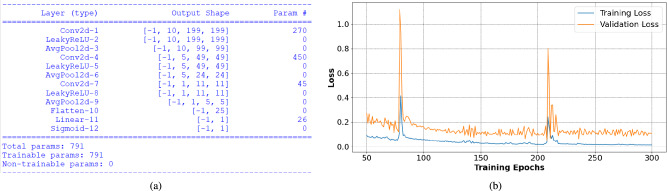
(**a**) Architecture of CNN-based classifier, and (**b**) training curve of CNN-based classifier.

**Network design and Training:** From the balanced dataset, $80\%$ of the data points are used to train the designed regression model, and the rest is used for testing and validation of the designed model. Before feeding the reconstructed images to the regression network, the images are resized to $400 \times 400$. The designed network architecture is shown in Fig. 9. Training is run for 300 epochs with the Adam optimizer and the binary cross-entropy loss function. The training curve is demonstrated in Fig. 9. The test set is constructed from the original dataset (before balancing by ADASYN), and it contains 28 data points. The accuracy over the test set is 92.8%.

#### CNN model for tumor size prediction

The aim of this model is to utilize the reconstructed S-parameter images to predict the size of lung tumors. However, more accurate size prediction is expected if the tumor is situated very closely to the plane of the antenna belt.

**Fig. 10:**
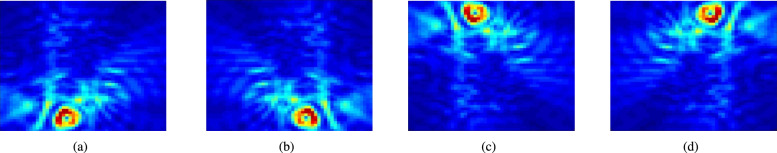
Different augmentation schemes on dataset: (**a**) Original image, (**b**) Horizontal flip, (**c**) Vertical flip, (**d**) $180^{\circ }$ rotation.

**Data augmentation:** The input simulation data has a total of 140 reconstructed S-parameter images (Simulation A), 70 simulations for each lung, where tumor size varies from 2 mm to 60 mm in diameter. For a particular tumor size, there are only two input images (one from each lung), which is insufficient for training the CNN-based model and may cause overfitting of the designed model. Hence, an appropriate data augmentation technique is employed to artificially increase the dataset size and generate multiple instances corresponding to each tumor size. In this work, very simple and straightforward augmentation techniques are employed, that are:1. Horizontal flip: where the image is flipped across the vertical axis; 2. Vertical flip: where the image is flipped across the horizontal axis; 3. 180 degree rotation: where the image is gone through both horizontal and vertical flip. The effect of various augmentation techniques is presented in Fig. 10. Hence, after employing the augmentation, the dataset size increases four times of the original size, where there are 8 instances for each tumor size (4 samples for each lung).

**Fig. 11:**
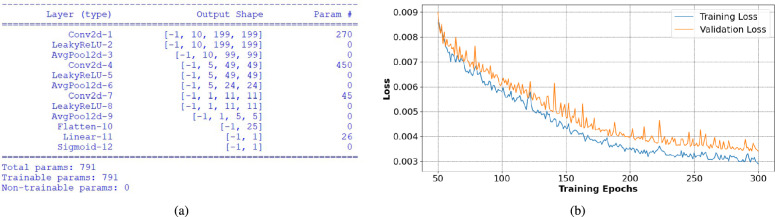
CNN model: (**a**) architecture for tumor size prediction, and (**b**) Learning curve for tumor size prediction.

**Model and training:** A convolution neural network-based regression model is developed to predict the tumor size given the reconstructed S-parameter images. The network is trained with the augmented dataset. The details of the CNN architecture are shown in Fig. 11. The designed network is very simple, with a very low number of trainable weights to avoid the risk of overfitting the model. Like the previous case, from the original dataset, 10 data points are taken out and are considered to be the test set used to measure the designed network’s performance (these are the same instances that were separated out during the XGBoost model training). The augmented versions of the corresponding instances have also been removed from the training set. This is because, if instances from the test set are seen beforehand during training, the estimation of accuracy will be biased. Hence, the remaining 520 instances comprise the training set, which is then again segregated into training (80%) and validation (20%) sets. The network is trained with Mean Squared Error (MSE) loss and Adam optimizer. The training is run for 300 epochs, after which the training and validation losses don’t improve. The learning curve is shown in Fig. 11 (b).

**Performance on the test set:** The trained network is fed with the test set, containing 10 instances. These instances are completely new to the trained model. The actual tumor radius and predicted tumor radius are shown in Table 2. All the predicted values are sufficiently close to the actual values. The mean square error for the test samples is 0.58 mm. The two-sided two-tail **t-test** is performed over the actual and predicted values of test data. We observe that the two-tailed P value equals 0.9495, which denotes that the difference between the two distributions is not statistically significant.

### Comparative discussion and ultimate model selection

**Fig. 12:**
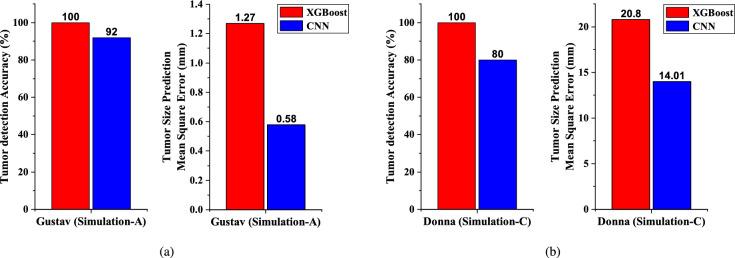
Performance comparison between different ML models for (**a**) Simulation-A, and (**b**) Simulation-C.

1. Over the Gustav body-model simulation data, i.e., Simulation-A, the XGBoost classifier on the S-parameter data gives better accuracy than the CNN-based classifier on reconstructed MW images for tumor detection. Whereas, for the size-prediction task, the CNN-based regression model performs better than the XGBoost regressor (as can be seen in Fig. 12).
2. The CNN-based regression model works on the reconstructed microwave images. Image data offers many methods for data augmentation, like rotation, flip, etc, which can not be applied over the raw S-parameters. Hence, the effective size of the training dataset can be increased by augmentation in the case of image data. This dataset augmentation is not applicable to the case of collected raw S-parameters. Thus, the CNN-based regression model is trained over a larger dataset as compared to the XGBoost model for the same collected dataset. For this reason, CNNs offer better generalization than the XGBoost model in the regression task for tumor size prediction.

Hence, in the actual workflow, as shown in Fig. 4 (a), the XGBoost-based classifier on raw S-parameters is chosen for the detection of tumors, and the CNN-based regression model on MW images is selected for tumor size prediction

### Fine-tuning the designed ML models for robustness against Noise

The aforementioned XGBoost classifier and CNN-based regression models have been trained on a simulated dataset, which is noiseless. However, in the actual scenario, noise is unavoidable. The added noise alters the actual data and may affect the performance of the ML models. One way to eliminate the problem is to make them robust against noise by fine-tuning them with an additional noisy version of the simulation dataset. During the fine-tuning, the previously trained (on noiseless simulation data) models are taken, and they are passed through additional training iterations with the newly constructed noise-infused dataset. This will alter the parameters of the trained model and make it adept to the noisy data.

For constructing the training set during fine-tuning, $50\%$ datapoints from Simulation A are taken randomly, and this data-subset is infused with three different degrees of random noise. The three different noise levels alter each of the S parameter values of each data point by $1\%$, $5\%$, and $10\%$, respectively. The previously designed XGBoost classifier and the CNN-based regression model are once again trained with this noisy dataset. For XGBoost, this step accounts for *incremental learning*^39^. For the CNN, the training is conducted for 100 epochs, with Adam optimizer and MSE loss function^40^.

For estimation of the performance of the fine-tuned models in comparison to the naive models, an additional test set is generated by taking 20 datapoints from the subset of simulation A, which was not used for fine-tuning, and then each datapoint is altered by $10\%$ of its original value. In the tumor prediction task, the fine-tuned XGBoost model gives an accuracy of $90\%$ (making 18 correct predictions out of 20), whereas the naive XGBoost gives a much poorer accuracy of $65\%$ (making 13 correct predictions out of 20). For the size prediction task on the testset, the fine-tuned CNN gives an average mean-squared error of 2.1 mm, whereas for the naive regression model, the error is 5 mm.

Hence, it is seen that when tested with a noisy test dataset, the fine-tuned models perform significantly better than the naive model trained with the simulated dataset. Thus, this fine-tuning gives some safeguard against the measurement noise that is present in the actual scenario.

## Results and discussion

The methodology and its performance are demonstrated on a simulation set up with a particular body model (Gustav). To validate the applicability of the proposed method and designed ML models on previously unseen data coming from some other body model, the procedure is tested on the female body model (Donna) available in CST Suite. Several simulations were conducted on this particular body model, and as mentioned before, this set is referred to as Simulation-C.

The XGBoost classifier correctly predicts the presence of a tumor in all simulations (i.e., accuracy $100\%$), whereas the CNN-based tumor detector gives $80\%$ accuracy; reinforcing our choice of XGBoost classifier over the CNN-based classifier for tumor detection. For the tumor size prediction, the actual tumor size and the predictions made by the CNN-based regression model on reconstructed microwave images and by the XGBoost-based regression model on S-parameters are shown in Table 3. It is seen that the CNN-based model performs better, justifying our choice, and it gives sufficiently close predictions for most of the cases.

**Table 3:** Predicted tumor sizes of Donna body model by the XGBoost Regression model, CNN over intensity MW image.

**Actual Tumor Radius (mm)**	**XGBoost** Predicted Radius (mm)	**CNN on MWI** Predicted Radius (mm)
5.0 (Left)	11.87	6.45
5.0 (Right)	10.85	5.79
10.0 (Left)	13.01	9.53
10.0 (Right)	13.26	10.7
15.0 (Left)	13.04	11.0
15.0 (Right)	17.53	10.8
20.0 (Left)	18.63	11.8
20.0 (Right)	12.7	22.8

The performance can be improved by training the model with different types of unseen data.As already demonstrated in the previous section, that the models can be made robust against noise by fine-tuning with noisy dataset. Similarly, this fine-tuning can also be performed when real-life data is available, during the trial process.

The full measurement and diagnosis procedure, which includes equipment setup, antenna calibration, data acquisition, and image processing, may typically require about 30–40 minutes. Specifically, setting up the VNA and switching matrix takes around 5–10 minutes, antenna calibration takes about 10 minutes, data collection varies between 30 seconds and 10 minutes depending on the equipment configuration and multiple readings, and data processing takes an additional 3–5 minutes. However, if the antennas are already calibrated and the VNA is pre-warmed, the measurement time in a clinical setting can be reduced to about 15 to 20 minutes. This makes it a safe and competitive option compared to a chest X-ray (which takes about 10–15 minutes) or a CT scan (about 15–30 minutes). In the future, automation and integrated systems can further minimize setup and calibration time, making the process faster and more suitable for routine clinical use, especially in the case of recurrent tumors.

## Conclusion

In this study, the challenge of early and recurring lung tumor detection has been addressed by employing a combination of microwave imaging and machine learning techniques. The proposed approach involves the design of an eight-antenna system integrated into a wearable belt, which is used to collect microwave scattering parameters and reconstruct microwave images. A suitable wideband, high-gain antenna is employed for this application. The system employs two machine learning models: an XGBoost classifier to predict tumor presence and a Convolutional Neural Network (CNN) to estimate tumor size, aiding in cancer staging. Initial testing was performed with simulation data from a CST body model of a 38-year-old male (‘Gustav’) and further validated with an independent dataset from a CST model of a 40-year-old female (‘Donna’), demonstrating the system’s robustness and adaptability. To ensure practical use, both models were fine-tuned to handle realistic noisy data, resulting in significantly improved performance compared to models trained only on clean simulation data. This fine-tuning provides resilience against measurement noise commonly encountered in clinical environments.

The key innovation of this work is the integration of a wearable microwave imaging system with machine learning to enable reliable and frequent monitoring of lung tumors. This approach shows strong potential for improving early detection and follow-up of recurrent lung cancer, which remains a critical health concern worldwide. Future research will focus on clinical trials with human subjects, further refinement to handle individual differences, and technical advancements to reduce overall measurement time, making the system more practical for real-world clinical workflows.

## Data Availability

The data sets generated and analyzed during the current study are available from the corresponding author on a reasonable request.
